# Efficacy of Anti-VEGF and Laser Photocoagulation in the Treatment of Visual Impairment due to Diabetic Macular Edema: A Systematic Review and Network Meta-Analysis

**DOI:** 10.1371/journal.pone.0102309

**Published:** 2014-07-16

**Authors:** Stephane Régnier, William Malcolm, Felicity Allen, Jonathan Wright, Vladimir Bezlyak

**Affiliations:** 1 Novartis Pharma AG, Basel, Switzerland; 2 Novartis Pharmaceuticals UK Ltd, Frimley Business Park, Frimley, Surrey, United Kingdom; 3 Numerus Ltd, Wokingham, United Kingdom; Medical University Graz, Austria

## Abstract

**Objective:**

Compare the efficacy of ranibizumab, aflibercept, laser, and sham in the first-line treatment of diabetic macular edema (DME) to inform technology assessments such as those conducted by the UK National Institute for Health and Care Excellence (NICE).

**Data sources:**

MEDLINE, Embase, Cochrane Library, congress abstracts, ClinicalTrials.gov registry and Novartis data on file.

**Inclusion criteria:**

Studies reporting 6- or 12-month results of randomized controlled trials (RCTs) evaluating at least two of ranibizumab 0.5 mg *pro re nata*, aflibercept 2.0 mg bi-monthly, laser photocoagulation or sham. Study quality was assessed based on likelihood of bias in selection, attrition, detection and performance.

**Outcome measure:**

Improvement in best-corrected visual acuity (BCVA) measured as the proportion of patients gaining ≥10 letters on the Early Treatment Diabetic Retinopathy Study scale. The outcome was chosen following acceptance by NICE of a Markov model with 10-letter health states in the assessment of ranibizumab for DME.

**Meta-analysis:**

Bayesian network meta-analyses with fixed and random effects adjusted for differences in baseline BCVA or central retinal thickness.

**Results:**

The analysis included 1,978 patients from eight RCTs. The random effects model adjusting for baseline BCVA was the best model based on total residual. The efficacy of ranibizumab was numerically, but not statistically, superior to aflibercept (odds ratio [OR] 1.59; 95% credible interval [CrI], 0.61–5.37). Ranibizumab and aflibercept were statistically superior to laser monotherapy with ORs of 5.50 (2.73–13.16) and 3.45 (1.62–6.84) respectively. The probability that ranibizumab is the most efficacious treatment was 73% compared with 14% for aflibercept, 12% for ranibizumab plus laser, and 0% for laser.

**Limitations:**

Three of the eight RCTs included are not yet published. The models did not adjust for all potential effect modifiers.

**Conclusion:**

Ranibizumab was non-significantly superior to aflibercept and both anti-VEGF therapies had statistically superior efficacy to laser.

## Introduction

Diabetic retinopathy is one of the main ocular complications associated with diabetes mellitus (DM). Diabetic macular edema (DME) is a frequent manifestation of diabetic retinopathy characterized by swelling of the retina due to fluid leakage from the blood vessels within the macula [1], [2], [3]. DME can lead to visual impairment (VI) and, if left untreated, blindness, as well as to reductions in productivity and health-related quality of life, resulting in substantial socioeconomic burden on the community [4], [5], [6]. The cross-sectional, observational Pan-European Prevalence Assessment of Diabetic Macular Edema with Visual Impairment (PREVAIL) study of patients with DM in seven European countries, concluded that VI due to DME is a significant complication of DM [7]. The overall prevalence of DME in the PREVAIL study was 5.4% and the overall prevalence of VI was 1.9% [7]. The prevalence of DM is increasing worldwide, with estimates indicating that DM affected 285 million adults (6.4% of the worldwide population) in 2010 [8]. This figure is projected to increase to 439 million (7.7%) by 2030 [8], thus the prevalence of DME and VI due to DME is expected to increase substantially over time.

Historically, treatment options for patients with VI due to DME were limited to non-pharmacological interventions, but management options for patients have expanded in recent years. Given the substantial burden of VI due to DME and the evolving options and clinical evidence for treatment, it is important to regularly compare the relative efficacy of available therapies. This study compares the relative efficacy of available first-line therapies that have available data. Before the availability of anti-vascular endothelial growth factor (anti-VEGF) therapy, laser photocoagulation therapy was the standard of care, providing vision stabilization in patients with DME, but with limited efficacy in providing clinically significant improvements in vision [9], [10], [11], [12], [13], [14]. Anti-VEGF therapy is the current standard of care [15]. Ranibizumab (Lucentis) is a monoclonal anti-VEGF-A antibody fragment administered as intravitreal (IVT) injections and was the first drug therapy to receive approval for the treatment of VI due to DME [16], [17]. A second anti-VEGF agent, aflibercept (Eylea), was submitted for European Union marketing authorization on 7 November 2013 [18]. The efficacy and safety of the pegylated anti-VEGF aptamer pegaptanib (Macugen) in the treatment of VI due to DME was investigated in phase II and III studies,[19], [20], [21] but the United Kingdom licence application was withdrawn in 2011 and it is understood that an application for a licence will no longer be pursued [22]. Bevacizumab (Avastin), a full-length anti-VEGF-A antibody developed for the treatment of cancer, has not been developed or licensed for IVT use and is therefore excluded from this analysis. This is consistent with guidance provided by the United Kingdom National Institute for Health and Care Excellence (NICE), which states that they “could not consider a comparison of ranibizumab with bevacizumab” and that “evidence, in particular about the balance of harms and benefits associated with bevacizumab, was not readily available for people with diabetic macular oedema” [23]. IVT triamcinolone (TA), a synthetic glucocorticoid, is not licensed for the treatment of DME [24]. IVT TA is not considered a comparator of routine use in the appraisal of anti-VEGF therapy by NICE and is therefore not considered relevant to this analysis [25]. Fluocinolone acetonide (FA) IVT implant (Iluvien) is approved in Europe only as a second-line therapy (for the treatment of VI associated with chronic DME, considered insufficiently responsive to available therapies) and therefore is not considered relevant to this analysis [26].

Several recent reviews have provided synopses of these therapies for DME and the relevant randomized controlled trials (RCTs) [15], [27], [28]. In addition, several recent systematic reviews (SRs) have compared RCT results for various treatment comparisons, concluding that anti-VEGF therapies consistently demonstrated efficacy superior to that of alternative therapies [29], [30], [31], [32], [33], [34], [35], [36]. Three of these studies presented conventional pair-wise meta-analyses [29], [31], [33], but none included a network meta-analysis and none compared all potential first line therapies (ranibizumab, aflibercept, and laser photocoagulation). This analysis compares the reported efficacy of laser photocoagulation therapy, ranibizumab IVT injection, ranibizumab IVT injection plus laser, aflibercept IVT injection and sham injections plus rescue laser therapy, within a network meta-analysis framework [37]. Moreover this analysis updates the current state of evidence by incorporating data from two large pivotal phase III RCTs (VIVID/VISTA) for aflibercept in DME. As such, this work is of importance to treatment and resource allocation decisions including technology appraisals such as those conducted by NICE in the UK. Finally, the method adjusts for effect modifiers such as baseline best-corrected visual acuity (BCVA) and central retinal thickness (CRT) to account for differences among study populations. Inclusion of appropriate disease modifiers is critical in conducting robust network meta-analyses [38].

## Methods

### Searches and data extraction

A systematic literature search was performed to identify relevant RCTs evaluating the efficacy of laser photocoagulation therapy, ranibizumab IVT injection, aflibercept IVT injection, ranibizumab plus laser or sham injection plus rescue laser therapy in the treatment of VI due to DME. The outcome of interest was efficacy, assessed as the percentage of patients achieving a gain in BCVA of at least 10 letters (2 lines) on the Early Treatment Diabetic Retinopathy Study (ETDRS) scale. The outcome of interest was chosen following acceptance by NICE of a Markov model with 10-letter health states in the assessment of ranibizumab for DME [23]. Changes in BCVA of 10 letters have been shown to be clinically significant in a number of studies [39], [40], [41], [42], [43].

The wealth of literature on the treatment of DME made a *de novo* SR impractical. To manage the volume of literature anticipated the search was conducted in three phases. In Phase 1, recently published, relevant SRs with a low risk of bias were identified through searches of the electronic databases Embase, MEDLINE, MEDLINE In-Process and the Cochrane Library. In Phase 2, an additional search was conducted to identify any relevant RCTs published since the most recent identified SR. The third phase involved hand searching of abstracts from ophthalmology congresses (Association for Research in Vision and Ophthalmology [ARVO], American Academy of Ophthalmology [AAO] and European Society of Retina Specialists [EURETINA]), the ClinicalTrials.gov registry, and data on file at Novartis.

A search strategy was developed for Embase using Medical Subject Headings and free-text search terms for DME and/or describing the treatments of interest (ranibizumab, aflibercept, laser and sham). This search was modified for MEDLINE and The Cochrane Library. A SR search filter with no date limit was included in Phase 1 (Table S1) [44]. A RCT filter and a 2012–present date (13 February 2014) limit was included in Phase 2, similar to the most recent identified SR (Table S2) [30].

A systematic reviewer (FA) conducted the database searches on 13th February 2014. Search results were downloaded into Endnote reference management software, which was used to manage the screening process. Inclusion and exclusion criteria were defined before screening the retrieved sources. To be included, studies had to be RCTs that reported the outcome patients achieving a gain in BCVA of at least 10 letters (2 lines) on the ETDRS scale for at least two comparators of interest (sham injections plus rescue laser, ranibizumab 0.5 mg *pro re nata* [as needed], ranibizumab 0.5 mg *pro re nata* plus laser, aflibercept 2.0 mg bi-monthly [every 2 months] and prompt laser photocoagulation therapy), and therefore studies with single treatment arms were excluded. The outcome of interest had to be measured at 6 or 12 months from study baseline, with 12 month data used for the analysis where available. Studies focusing on a specific ethnic group were not included in the base-case analysis but were included in the sensitivity analyses. Studies published in English, French and German were included. Two authors (SR and FA) independently assessed the eligibility of all retrieved sources based on published abstracts. Non-relevant papers were excluded with the reasons for exclusion documented using a prospectively designed coding system. Discrepancies were resolved through discussion. Inclusion or exclusion of potentially relevant full-text RCT publications was then verified by three authors (FA, SR, WM) through a full text review. Study characteristics and outcome data including baseline characteristics, number of patients, country, key inclusion and exclusion criteria and quality appraisal were captured in a data extraction table in Microsoft Excel. Data were extracted by two authors (SR and WM).

### Study quality assessment

The quality of, and risk of bias associated with, the methodology of each SR was assessed by two authors (SR and FA) using the Scottish Intercollegiate Guidelines Network tool [44]. The tool allows critical elements of the study design and results to be rated as: well covered, adequately addressed, not addressed, not reported, or not applicable. For SRs to be included, they had to have an appropriate and clearly focussed study question, a clear description of the methodology, sufficiently rigorous literature searches (including MEDLINE, Embase, The Cochrane Library and hand-searching of reference lists), and assessment of the quality of included data sources [44]. The quality of each RCT was assessed according to the methodology checklist detailed in Appendix C of the NICE Guidelines Manual 2012 [45]. In brief, we assessed the likelihood of bias in selection, attrition, detection and performance. Two authors (SR and FA) independently assessed the quality of the selected studies, with discrepancies were resolved through discussion.

### Network meta-analyses

To evaluate the relative efficacy of the interventions of interest, we conducted Bayesian network meta-analyses with fixed and random treatment effects (Information S1). To estimate the posterior distribution for each model, two Markov chain Monte Carlo (MCMC) simulations were run for 20,000 iterations each. Results are reported after excluding the first 2,000 iterations. The convergence of each chain was assessed using Brooks-Gelman-Rubin (BGR) diagnostic plots. Convergence was diagnosed when the ratio of between- and within-chain variance was close to 1, and the lines representing within- and between-chain variability converged and were stable.[46], [47] The relative treatment effect was the odds ratio (OR) for the percentage of patients experiencing an improvement in BCVA of at least 10 letters on the ETDRS scale. A statistically significant OR above 1 indicates superiority of an intervention over its comparators. The overall relative treatment effect was calculated using the median value from the posterior distribution. A 95% credible interval (CrI) was created using the 2.5 and 97.5 percentiles of the posterior distribution. An OR was considered statistically significant at the 5% level if the 95% CrI did not contain 1. Similar to Ford *et al*. [29], a relative treatment effect was interpreted as non-significant if the 95% CrI for the OR crossed 1. Analyses were performed (by SR and WM) using WinBUGS version 1.4 (MRC Biostatistics Unit, Cambridge, United Kingdom) and results were independently validated by a second author (JW) using a Bayesian algorithm in SAS version 9.4 (SAS Institute Inc., Cary, NC, USA).

### Sensitivity analyses

To test the sensitivity of the results to the systematic review and meta-analysis methodology, a number of sensitivity analyses were conducted. Baseline BCVA and CRT have been shown to have an effect on the level of improvements in BCVA in patients treated with anti-VEGF for DME and NICE considers baseline CRT an important clinical variable in the treatment of DME [23], [48]. Therefore, the present analysis included baseline BCVA and/or CRT as covariates in the models. A number of candidate models were constructed with either fixed or random treatment effects and including as covariates baseline BCVA, baseline CRT, both BCVA and CRT, or neither parameter. The interaction coefficients between treatment and covariates were assumed to be the same for all treatment arms in the base-case analysis, but were varied in sensitivity analyses. First, the assumption of exchangeable coefficients across treatments was tested (i.e. the coefficient of baseline BCVA was drawn from a normal distribution rather than being a constant value). Second, to capture the potential impact of baseline BCVA differing by treatment, the effect of baseline BCVA was allowed to vary between anti-VEGF treatment and other treatments (laser or sham). The candidate models were assessed using the deviance information criterion (DIC), total residual deviance and available evidence in the literature. More specifically, the posterior mean of the total residual deviance was compared with the number of unconstrained points [49]. A difference in DIC of more than 3–5 points between two models was considered significant and the model with the lowest DIC was elected the better model [50]. If the difference in DIC between two models was 3 points or less, the authors used the total residual deviance and the clinical evidence published in the literature to elect the better model.

Additionally, the effect of including the studies that were excluded during the screening of the systematic review was assessed (i.e. studies focussing on a specific ethnic group). Some studies in our base case included treatment arms that were not of interest (e.g. IVT TA). The impact of including such arms was also estimated.

Finally, a node-splitting analysis was used to compare the direct and indirect evidence for the laser and the ranibizumab plus laser estimates. Note that there was only one closed loop in the network meta-analysis (ranibizumab monotherapy, ranibizumab plus laser, laser monotherapy) and direct versus indirect evidence could only be assessed against those treatments.

## Results

### Systematic review

In Phase 1, electronic database searches retrieved 183 citations for screening (MEDLINE, 43; Embase, 104; The Cochrane Library, 36) of which eight SRs were reviewed for inclusion [29], [30], [32], [33], [34], [35], [36], [51]. After evaluation of the full text, four SRs were excluded, three because they only reported on one treatment of interest [29], [34], [35] and one because it focussed on health utilities and did not report the outcome of interest [51]. The remaining four SRs were included in the review [30], [32], [33], [36]. The included SRs reported data from 12 potentially relevant RCTs, nine with ranibizumab and laser/sham treatment arms (READ-2, READ-3, RESOLVE, RESTORE, DRCR.net Protocol I, RIDE, RISE, REVEAL and RETAIN) and three with aflibercept and laser treatment arms (DA VINCI, VIVID and VISTA).

In Phase 2, electronic database searches retrieved 376 citations for screening (MEDLINE, 115; Embase, 246; The Cochrane Library, 15) and screened for potentially relevant RCTs. Seven publications were reviewed in full, but all were excluded because they did not report the outcome of interest at 6 or 12 months (Table S3) [52], [53], [54], [55], [56], [57], [58].

In Phase 3, the search of the ClinicalTrials.gov registry identified records of a further five completed RCTs with ranibizumab and laser/sham treatment arms (RESPOND, RED-ES, RELIGHT, OPTIMAL and RaScaL) [59], [60], [61], [62], [63] and no further RCTs with aflibercept and laser/sham treatment arms. The search of ophthalmology congress abstracts did not identify any additional relevant RCTs.

In total, 17 RCTs were identified, 12 from electronic database searches and a further five from hand-searching of the ClinicalTrials.gov registry. Of these, seven were excluded because they included either no or only one regimen of interest (RELIGHT, RETAIN, READ-3, RaScaL, OPTIMAL, RIDE and RISE), one (RED-ES) was excluded as it did not have any available results, and one (REVEAL) was excluded from the base-case analysis (but included in sensitivity analyses) because the focus was on a single ethnic group (Asian). In READ-2, patients received four ranibizumab injections in the first 6 months (at baseline and months 1, 3 and 5) independent of visual acuity or disease progression, rather than treatment *pro re nata*. This was lower than the mean number of injections (4.8 in the first 6 months) received in RESTORE. Therefore, READ-2 was included in the base-case analysis as a conservative measure and the impact of this inclusion was investigated in sensitivity analyses.

**Figure 1:**
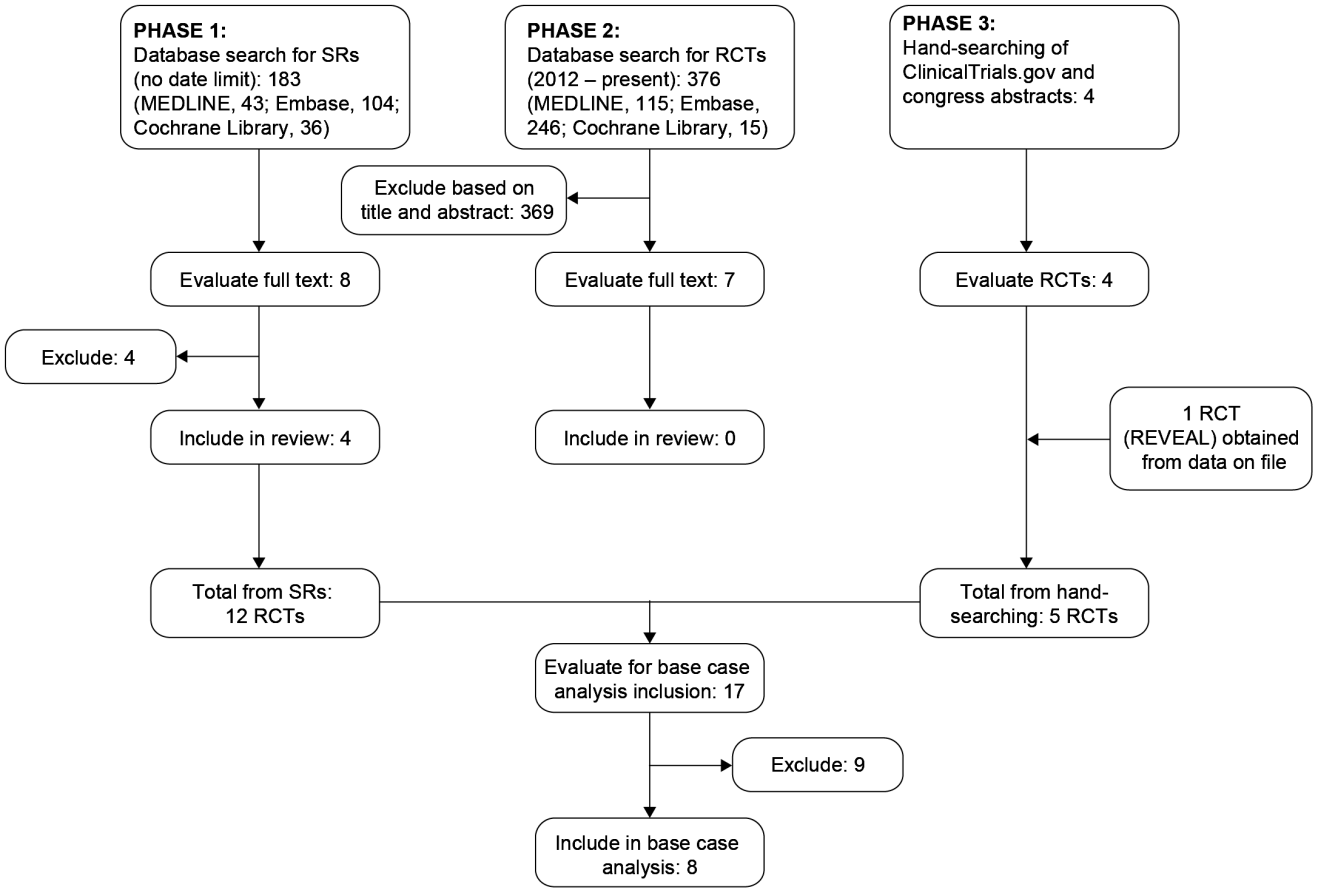
PRISMA diagram of the screening and selection process.

Overall, eight RCTs were included in the base-case analysis (VIVID, VISTA, DA VINCI, RESTORE, READ-2, RESOLVE, RESPOND, DRCR.net Protocol I) [48], [62], [64], [65], [66], [67], [68], [69]. Outcomes were reported at month 12 in all studies except READ-2, for which outcomes were reported at month 6. A Preferred Reporting Items for Systematic Reviews and Meta-Analyses (PRISMA) diagram showing the screening and selection process is presented in Figure 1 [70].

### Study quality assessment

The four included SRs were judged to have a low risk of bias through quality assessment (Table S4) [30], [32], [33], [36]. All four SRs reported robust methodology and conducted quality appraisals of included RCTs. The quality of the included RCTs varied as three of the included trials were not yet published in peer-reviewed journals (data on file for RESPOND, congress presentation for VIVID and VISTA). Overall the studies were of good quality, although some issues were identified (Table S5). In READ-2, the use of masking was not clearly reported and it was unclear whether the reported sample size was based on the full analysis set (all randomized patients with baseline assessments, at least one post baseline assessment, and who received any study treatment). To be conservative, the number of patients randomized to each treatment in READ-2 was used in the analysis. Randomization and blinding were also unclear. In DRCR.net Protocol I, blinding was broken at month 6 in the ranibizumab plus deferred laser treatment arm, although this arm was not included in the base-case analysis.

### Study network

**Figure 2:**
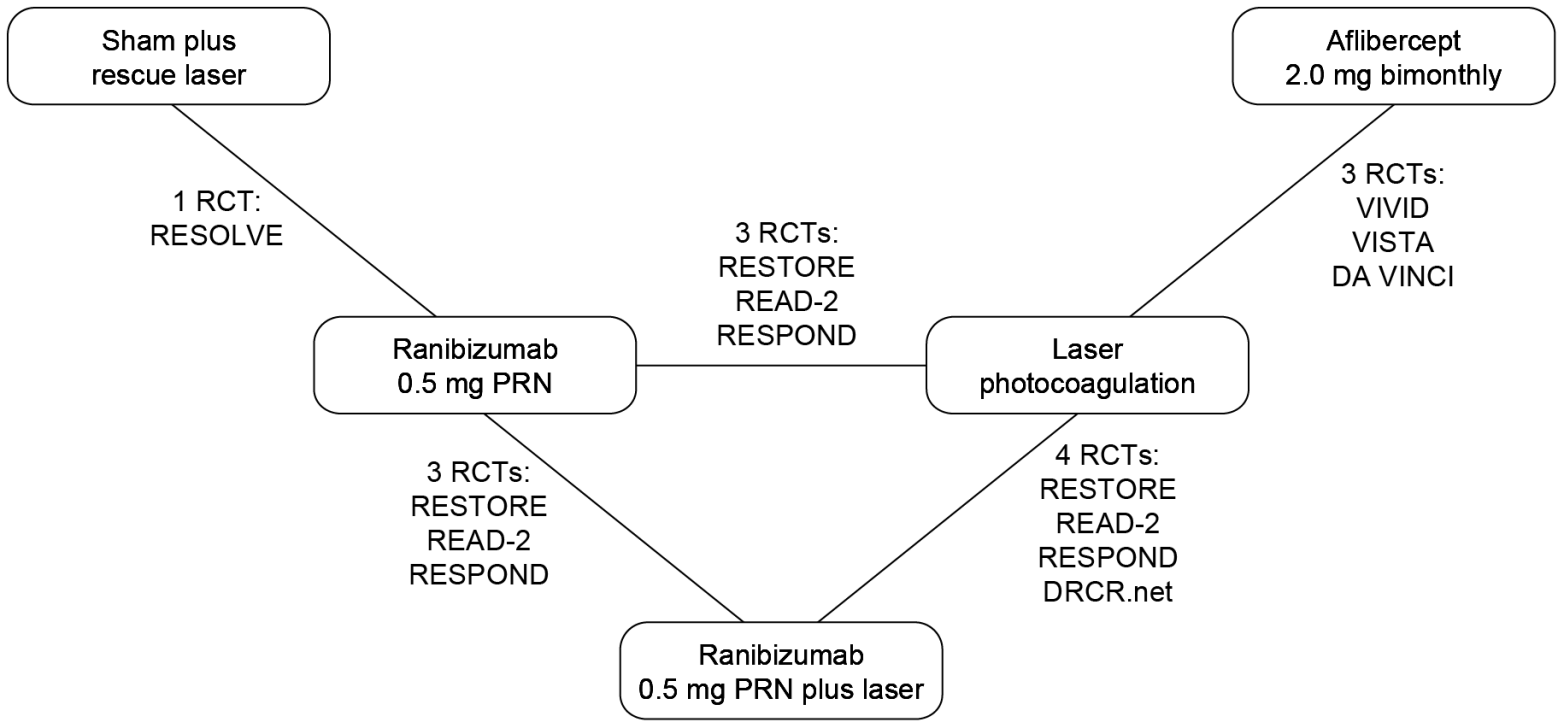
Study network.

The study network showing the possible treatment comparisons based on the available data from the eight included RCTs is presented in Figure 2. The included studies represented 1,978 patients (847 patients treated with laser, 334 treated with ranibizumab, 328 treated with aflibercept, 420 treated with ranibizumab plus laser, and 49 receiving sham treatment). The numbers of patients experiencing an improvement in BCVA of at least 10 letters on the ETDRS scale in the selected RCTs are summarized in Table 1. Baseline BCVA and CRT of patient populations varied among the included RCTs (Table S6 and Table S7). The inclusion criterion for baseline BCVA in the VIVID and VISTA RCTs was 24–73 letters compared with 39–78 letters in RESTORE. There was only one closed loop in the network meta-analysis (ranibizumab monotherapy, ranibizumab plus laser, laser monotherapy) and direct versus indirect evidence could only be assessed against those treatments.

**Table 1:** Summary of the proportion of patients gaining ≥10 letters (BCVA) from baseline to month 12 by study and treatment group.

		Treatment group				
Study	Outcome measured at	Ranibizumab 0.5 mg PRN, n/N (%)	Aflibercept 2.0 mg bi-monthly, n/N (%)	Laser, n/N (%)	Sham, n/N (%)	Ranibizumab 0.5 mg PRN + laser, n/N (%)
DA VINCI [66]	12 months		19/42 (45.2)	13/44 (29.5)		
VISTA[72]	12 months		88/151 (58.3)	30/154 (19.5)		
VIVID[72]	12 months		72/135 (53.3)	34/132 (25.8)		
DRCR.net Protocol I [67]	12 months			81/293 (27.6)		95/187 (50.8)
READ-2[69] *	6 months	17/42 (40.5)		2/42 (4.8)		12/42 (28.6)
RESOLVE [68]	12 months	62/102 (60.8)			9/49 (18.4)	
RESPOND [73]	12 months	37/75 (49.3)		10/72 (13.9)		24/73 (32.9)
RESTORE [48]	12 months	43/115 (37.4)		17/110 (15.5)		51/118 (43.2)
Total		159/334 (47.6)	179/328 (54.6)	187/847 (22.1)	9/49 (18.4)	182/420 (43.3)

Not all treatment groups included in the network meta-analysis are presented. Analysis is based on the intention-to-treat population of each study.

*Data for READ-2 are proportion of patients who gained ≥10 letters (BCVA) from baseline to month 6. After 6 months, patients in the laser treatment arm could receive ranibizumab, preventing meaningful comparisons between treatment arms at 12 months.

BCVA, best-corrected visual acuity; bimonthly, every 2 months; n, number of patients in treatment arm with ≥10 letter increase in BCVA; N, total number of patients in study treatment arm; PRN, *pro re nata* (as needed).

### Network meta-analysis results

The pairwise ORs of the network meta-analysis with random treatment effects and baseline BVCA included as a covariate are presented in Table 2. The pairwise ORs indicate the relative treatment effect; a statistically significant OR above 1 indicates superiority of an intervention over its comparators. The efficacy of ranibizumab 0.5 mg monotherapy was numerically, but not statistically, superior to aflibercept monotherapy (OR, 1.59; 95% CrI, 0.61–5.37). Ranibizumab and aflibercept monotherapies had a statistically higher efficacy than laser with ORs of 5.50 (95% CrI, 2.73–13.16) and 3.45 (95% CrI, 1.62–6.84), respectively. The efficacy of ranibizumab monotherapy was statistically superior to sham (OR, 6.50; 95% CrI, 1.66–24.99). The efficacy of aflibercept monotherapy was numerically superior to sham, although the OR was not statistically significant (OR, 4.06; 95% CrI, 0.60–21.84). Based on the random treatment effects model with baseline BCVA included as a covariate, the proportion of patients gaining at least 10 letters was 54% (95% CrI, 38–72) for ranibizumab monotherapy, 43% (95% CrI, 25–59) for aflibercept and 18% (95% CrI, 14–21) for laser monotherapy. Table 3 shows the probability that an intervention is the most effective treatment in the network. Ranibizumab monotherapy had the highest probability (73%) of being the most effective treatment in the network (aflibercept, 14%; ranibizumab plus laser, 12%; laser, 0%).

**Table 2:** Pair-wise odds ratios (95% credible intervals) from the random treatment effects model with baseline BVCA included as a covariate.

	Laser	Ranibizumab 0.5 mg PRN	Aflibercept 2.0 mg bi-monthly	Ranibizumab 0.5 mg + laser	Sham
**Laser**	–				
**Ranibizumab 0.5 mg PRN**	5.50 (2.73–13.16)*	–	1.59 (0.61–5.37)		6.50 (1.66–24.99)*
**Aflibercept 2.0 mg bi-monthly**	3.45 (1.62–6.84)*	0.63 (0.19–1.63)	–		4.06 (0.60–21.84)
**Ranibizumab + laser**	4.05 (2.16–8.65)*	0.74 (0.35–1.46)	1.18 (0.45–3.66)	–	
**Sham**	0.85 (0.19–4.56)	0.15 (0.04–0.60)*	0.25 (0.05–1.65)	0.21 (0.05–1.01)	–

*p<.05.

Pair-wise odds ratios indicate the relative treatment effect for the treatments compared in the network meta-analysis. A statistically significant odds ratio greater than 1 indicates that the treatment in the corresponding row is superior to the treatment in the corresponding column.

BCVA, best-corrected visual acuity; PRN; *pro re nata* (as needed).

**Table 3:** Probability that each treatment is the most efficacious treatment in the network.

Treatment	Probability best treatment
Laser	0%
**Ranibizumab 0.5 mg PRN**	**73%**
Aflibercept 2.0 mg bi-monthly	14%
Ranibizumab 0.5 mg PRN + laser	12%
Sham + rescue laser	1%

PRN; *pro re nata* (as needed).

The results for the fixed treatment effects model with baseline BCVA as a covariate were similar: the efficacy of ranibizumab was numerically, but not statistically significantly superior to aflibercept (OR, 1.49; 95% CrI, 0.80–2.78). Ranibizumab and aflibercept monotherapies remained statistically superior to laser monotherapy with ORs of 5.18 (95% CrI, 3.30–8.20) and 3.47 (95% CrI, 2.43–4.98) respectively. Based on the fixed treatment effect model, the probability that ranibizumab monotherapy is the best treatment in the network was 86% compared with 10% for aflibercept and 4% for ranibizumab plus laser.

### Model comparison (sensitivity analysis)

All tested models showed similar OR estimates between ranibizumab and aflibercept (range of point estimates, 1.30–1.66) (Table 4). Evaluation of the candidate models based on the DIC and the total residual deviance confirmed the selection of the random treatment effects model including baseline BCVA as a covariate as the best model (base-case) The DIC was comparable across models (range was less than 3 points). The base-case model had a low DIC (124.7) compared with the other model models tested and the posterior mean of the total residual deviance was 19.0 versus 19.0 unconstrained points, indicating a good fit for the base-case model. The choice of a random treatment effect model (over a fixed treatment effect) was justified because of the heterogeneity among trials (with a between-study standard deviation of 0.40).

**Table 4:** Model selection: comparison of the total residual deviance and DIC for Bayesian network meta-analyses with fixed or random treatment effects and baseline BCVA and/or CRT as covariates.

Treatment effect	Covariate adjustment	Interaction coefficient between treatment and covariates	OR (95% CrI)*	Total residual deviance (rank)	DIC (rank)
Fixed	BCVA at baseline	Same for all treatments	1.49 (0.80–2.80)	22.5 (7)	124.2 (1)
Random	None	None	1.30 (0.54–4.17)	19.6 (5)	124.6 (2)
**Random**	**BCVA at baseline**	**Same for all treatments**	1.59 (0.61–5.37)	19.0 (1)	124.7 (3)
	BCVA at baseline	Same for anti-VEGF and same for laser and sham + laser	1.30 (0.44–4.78)	19.3 (4)	125.3 (4)
		Exchangeable between treatments	1.54 (0.47–6.25)	19.2 (3)	125.5 (5)
**Random**	CRT at baseline	Same for all treatments	1.34 (0.51–4.94)	19.7 (6)	125.5 (5)
**Random**	BCVA & CRT at baseline	Same for all treatments	1.66 (0.53–6.72)	19.1 (2)	125.5 (5)

*Relative treatment effect for ranibizumab 0.5 mg PRN versus aflibercept 2.0 mg bi-monthly.

BCVA, best-corrected visual acuity; CrI, credible interval; CRT, central retinal thickness; DIC, deviance information criterion; OR, odds ratio; PRN; *pro re nata* (as needed); VEGF, vascular endothelial growth factor.

The coefficient reflecting the impact of baseline BCVA on the odds (log scale) of gaining at least 10 letters BCVA was −0.12 (95% CrI: −0.36 to 0.08). To assess the validity of this coefficient, we ran a logistic regression on RESTORE patient-level data with treatment and baseline BCVA. The resulting coefficient was −0.04 (95% confidence interval: −0.07 to −0.01), thus the credible interval from the network meta-analyses included the results from patient-level data.

There was no statistically significant inconsistency between direct and indirect evidence with the difference between estimates being 0.48 in favour of the direct estimates (95% CrI, −1.05 to 2.05, *p* = .42).

The REVEAL study was excluded from the base-case analysis because it compared ranibizumab, ranibizumab plus laser therapy, and laser therapy in an Asian population. In sensitivity analyses, inclusion of this study did not substantially modify the results; ranibizumab remained non-significantly superior to aflibercept (OR, 1.34; 95% CrI, 0.62–3.53). With the inclusion of REVEAL in the analysis, the probability that ranibizumab is the most efficacious treatment in the network was 62% versus 20% for aflibercept and 18% for ranibizumab plus laser. In a scenario excluding READ-2, the efficacy of ranibizumab monotherapy remained numerically, but not statistically significantly superior to aflibercept monotherapy (OR, 1.42; 95% CrI, 0.30–8.71). With the inclusion of the ranibizumab plus deferred laser arm from DRCR.net Protocol I as a ranibizumab treatment arm, ranibizumab monotherapy remained numerically, but not statistically, superior to aflibercept monotherapy (OR, 1.35; 95% CrI, 0.53–4.46). In this scenario, the probability that ranibizumab plus laser is the most efficacious treatment in the network increased from 12% to 24% (versus 54% for ranibizumab and 21% for aflibercept).

With the inclusion of monthly aflibercept treatment arms from VIVID, VISTA and DA VINCI as well as TA and TA plus laser treatment arms from DRCR.net Protocol I, ranibizumab remained numerically, but not statistically, superior to aflibercept bi-monthly (OR, 1.59; 95% CrI, 0.69–4.62). In this scenario, the 95% CrI was narrower than in the base-case analysis (0.61–5.37). For the scenario including monthly aflibercept, TA and TA plus laser, an independently run Bayesian model (using SAS version 9.4) gave similar results (OR, 1.54; 95% CrI, 0.65–4.17).

## Discussion

To our knowledge, this systematic review and network meta-analysis is the first to include phase III data for aflibercept. This is an important addition to the current literature because aflibercept was submitted for European Union approval in November 2013. Several recent systematic reviews of treatment options for VI due to DME have concluded that anti-VEGF therapies consistently demonstrated superior efficacy compared with alternative therapies, but none has compared approved (or soon to be approved) anti-VEGF therapies with laser photocoagulation in a comprehensive network meta-analysis [29], [30], [31], [32], [33], [34], [35], [36].

The results of the current systematic review and network meta-analysis confirm the findings of RCTs demonstrating superiority of anti-VEGF monotherapy over laser monotherapy in the treatment of VI due to DME [48], [66], [69]. In this analysis the anti-VEGF therapies ranibizumab and aflibercept had a statistically significantly higher efficacy than prompt laser monotherapy, and prompt laser therapy had a 0% probability of being the most efficacious treatment in the network. Furthermore, it cannot be proven that adding laser to ranibizumab provides additional benefits over anti-VEGF monotherapy; combination therapy had only a 12% probability of being the most efficacious treatment. Comparison of the efficacy of anti-VEGF therapies in all tested models indicated that the efficacy of ranibizumab monotherapy was numerically, but not statistically significantly, superior to aflibercept monotherapy.

There were differences between observed and modelled outcomes. Across the eight trials included in this analysis, the proportion of patients gaining at least 10 letters was modelled (adjusting for BCVA at baseline) to be 54% for ranibizumab monotherapy (95% CrI, 38–72), 43% for aflibercept (95% CrI, 25–59) and 18% for laser monotherapy (95% CrI, 14–21). For aflibercept, the observed unweighted mean proportion of patients gaining at least 10 letters was 52% (55% if weighted by trial size), substantially higher than the results from the model (43%). This may be due to higher proportions of patients responding across all treatment arms of the aflibercept RCTs. In VIVID, VISTA and DA VINCI, higher proportions of patients responded to laser therapy than in READ-2, RESPOND and RESTORE. Such differences in response to the same treatment among different RCTs may be the result of inter-trial heterogeneity in baseline characteristics, or may arise by chance. The Bayesian models in this analysis account for both effects. Based on the observed trial data, aflibercept therapy resulted in the highest proportion of patients gaining at least 10 letters, whereas in the model adjusting for baseline BCVA, the proportion of patients gaining at least 10 letters was highest with ranibizumab therapy. This highlights the importance of using robust methodologies that adjust for potential effect modifiers in comparisons of RCTs. Drawing conclusions based on raw data may be misleading.

The results of the analysis were robust to changes in the included RCTs and treatment arms. Including REVEAL, excluding READ-2 and including the ranibizumab plus deferred laser arm from DRCR.net Protocol I did not substantially modify the results: in all three scenarios, ranibizumab had the highest probability of being the most efficacious treatment in the network, although not significantly better than aflibercept bimonthly. In the base case, the probability that ranibizumab plus laser or aflibercept monotherapy are the most efficacious treatment in the network was 12% and 14% respectively. These probabilities increased slightly with the inclusion of REVEAL (18% for ranibizumab plus laser; 20% for aflibercept), the exclusion of READ-2 (26% for both treatments) and the inclusion of the ranibizumab plus deferred laser arm from DRCR.net Protocol I (24% for ranibizumab plus laser; 14% for aflibercept). With the inclusion of TA plus laser (DRCR.net Protocol I) and monthly aflibercept (VIVID, VISTA, DA VINCI), ranibizumab monotherapy remained numerically, but not statistically, superior to aflibercept monotherapy. The point estimate for the odds ratio of ranibizumab versus aflibercept did not change but the width of the 95% CrI decreased, most likely due to the additional information available.

A strength of the current analysis is the inclusion of baseline BCVA and CRT as covariates in the models to account for differences in baseline visual acuity and disease progression among the patient populations of different RCTs. In particular, baseline BCVA varied among patient populations: the inclusion criterion for BCVA in VIVID and VISTA was 24–73 letters compared with 39–78 letters in RESTORE. Adjusting for baseline visual acuity and disease progression among the patient populations is critical in comparisons among RCTs because gains in BCVA with anti-VEGF therapy in patients with DME and other retinal diseases such as neovascular (wet) age-related macular degeneration have been shown to be greater in patients with a worse baseline BCVA than in patients with a better BCVA.[48], [71] The coefficient reflecting the impact of baseline BCVA on the logarithm of the odds of gaining at least 10 letters BCVA (−0.12) was consistent with a negative correlation between baseline BCVA and gain in BCVA as a result of treatment. The random treatment effects model including baseline BCVA provided a good fit because the posterior mean of the total residual deviance was 19.0 versus 19.0 unconstrained points.

This study has some limitations. The analysis included a relatively small number of RCTs, of which three are not yet published in full and although the RCTs included in the meta-analysis were, in general, of good quality, the use of masking was not clearly reported in READ-2. Exclusion of READ-2 during sensitivity analyses suggested that this did not have a substantial effect on the results. Finally, the RESOLVE RCT was a dose finding study with starting doses of 0.3 and 0.5 mg; after month 1 there was no true 0.5 mg treatment arm as the dose could be doubled. Consistent with the RESOLVE publication, we used the pooled results in the analysis since they “are considered to be representative for treatment with 0.5-mg injections”[68].

Given the substantial burden of VI due to DME and the evolving options for treatment, it is important to regularly compare the relative efficacy of the available first-line therapies. This study is the first comparative network meta-analysis comparing anti-VEGF therapy with laser photocoagulation to include phase III data for aflibercept. Thus this analysis updates the state of evidence available to inform treatment and resource allocation decisions. In this study, ranibizumab was non-significantly superior to aflibercept and both anti-VEGF therapies were statistically superior to laser monotherapy.

## Supporting Information

Table S1 **Embase search strategy for systematic reviews (no date limit, 1974 to 13 February 2014).**
(DOCX)

Table S2 **Embase search strategy for randomized controlled trials published since 2012 (1996 to 13 February 2014).**
(DOCX)

Table S3 **Studies from randomized controlled trial database search excluded based on full text review.**
(DOCX)

Table S4 **Quality appraisal of included systematic reviews.**
(DOCX)

Table S5 **Quality appraisal of included randomized controlled trials.**
(DOCX)

Table S6 **Summary of baseline BCVA (ETDRS letter score) in the study eye by study and treatment group.**
(DOCX)

Table S7 **Summary of baseline CRT/CFT in the study eye by study and treatment group.**
(DOCX)

Checklist S1 PRISMA 2009 checklist.
(DOC)

Information S1 Fixed and random treatment effect model.
(DOCX)
