# Supplementary material for: Efficacy of Anti-VEGF and Laser Photocoagulation in the Treatment of Visual Impairment due to Diabetic Macular Edema: A Systematic Review and Network Meta-Analysis
Source: PLoS One. 2014 Jul 16;9(7):e102309. doi: 10.1371/journal.pone.0102309 (PMC4100770; doi:10.1371/journal.pone.0102309)
Supplement: Table S1 — Embase search strategy for systematic reviews (no date limit, 1974 to 13 February 2014). (DOCX) [file pone.0102309.s001.docx]

Table S1. Embase search strategy for systematic reviews (no date limit, 1974 to 13 February 2014).

|  | **Search term** | **Number of records** |
| --- | --- | --- |
| 1 | exp Macular Edema/ | 10,367 |
| 2 | exp Macula Lutea/ | 7,556 |
| 3 | (macula$ adj3 edema).mp. | 11,931 |
| 4 | (macula$ adj3 oedema).mp. | 1,508 |
| 5 | DMO.mp. | 541 |
| 6 | exp diabetic macular edema/ | 1,857 |
| 7 | exp diabetic retinopathy/ | 28,520 |
| 8 | diabetic maculopathy.mp. | 502 |
| 9 | (diabet$ adj2 macular adj (oedema or edema)).mp. | 2,996 |
| 10 | 1 or 2 or 3 or 4 or 5 or 6 or 7 or 8 or 9 | 44,494 |
| 11 | exp ranibizumab/ | 3,676 |
| 12 | lucentis.mp. | 1,588 |
| 13 | aflibercept.mp. | 1,443 |
| 14 | vegf trap-eye.mp. | 53 |
| 15 | eylea.mp. | 110 |
| 16 | exp aflibercept/ | 1,411 |
| 17 | sham.mp. | 74,695 |
| 18 | laser.mp. | 234,267 |
| 19 | 11 or 12 or 13 or 14 or 15 or 16 or 17 or 18 | 311,490 |
| 20 | 10 and 19 | 8,215 |
| 21 | Meta-Analysis as Topic/ | 11,740 |
| 22 | meta analy$.tw. | 78,683 |
| 23 | metaanaly$.tw. | 3,932 |
| 24 | Meta-Analysis/ | 80,674 |
| 25 | (systematic adj (review$1 or overview$1)).tw. | 64,858 |
| 26 | exp Review Literature as Topic/ | 47,281 |
| 27 | 21 or 22 or 23 or 24 or 25 or 26 | 206,843 |
| 28 | cochrane.ab. | 37,010 |
| 29 | medline.ab. | 69,003 |
| 30 | (psychlit or psyclit).ab. | 985 |
| 31 | (cinahl or cinhal).ab. | 11,438 |
| 32 | 28 or 29 or 30 or 31 | 53,169 |
| 33 | 27 or 32 | 222,614 |
| 34 | 20 and 33 | 104 |
