# Supplementary material for: Efficacy of Anti-VEGF and Laser Photocoagulation in the Treatment of Visual Impairment due to Diabetic Macular Edema: A Systematic Review and Network Meta-Analysis
Source: PLoS One. 2014 Jul 16;9(7):e102309. doi: 10.1371/journal.pone.0102309 (PMC4100770; doi:10.1371/journal.pone.0102309)
Supplement: Table S2 — Embase search strategy for randomized controlled trials published since 2012 (1996 to 13 February 2014). (DOCX) [file pone.0102309.s002.docx]

Table S2. Embase search strategy for randomized controlled trials published since 2012 (1996 to 13 February 2014).

|  | **Search term** | **Number of records** |
| --- | --- | --- |
| 1 | exp Macular Edema/ | 8,961 |
| 2 | exp Macula Lutea/ | 4,086 |
| 3 | (macula$ adj3 edema).mp. | 9,975 |
| 4 | (macula$ adj3 oedema).mp. | 1,258 |
| 5 | DMO.mp | 230 |
| 6 | exp diabetic macular edema/ | 1,857 |
| 7 | exp diabetic retinopathy/ | 19,518 |
| 8 | diabetic maculopathy.mp. | 346 |
| 9 | (diabet$ adj2 macular adj (oedema or edema)).mp. | 2,861 |
| 10 | 1 or 2 or 3 or 4 or 5 or 6 or 7 or 8 or 9 | 30,385 |
| 11 | exp ranibizumab/ | 3,673 |
| 12 | lucentis.mp. | 1,587 |
| 13 | aflibercept.mp. | 1,441 |
| 14 | vegf trap-eye.mp. | 53 |
| 15 | eylea.mp. | 110 |
| 16 | exp aflibercept/ | 1,409 |
| 17 | sham.mp. | 57,755 |
| 18 | laser.mp. | 185,419 |
| 19 | 11 or 12 or 13 or 14 or 15 or 16 or 17 or 18 | 245,814 |
| 20 | 10 and 19 | 5,823 |
| 21 | clinical trial/ | 731,445 |
| 22 | randomized controlled trial/ | 316,945 |
| 23 | exp randomization/ | 54,781 |
| 24 | single blind procedure/ | 17,610 |
| 25 | double blind procedure/ | 93,383 |
| 26 | crossover procedure/ | 35,617 |
| 27 | exp placebo/ | 187,944 |
| 28 | Randomi?ed controlled trial$.mp. | 395,367 |
| 29 | RCT.mp. | 14,065 |
| 30 | Random allocation.mp. | 1,123 |
| 31 | Randomly allocated.mp. | 16,421 |
| 32 | allocated randomly.mp. | 1,306 |
| 33 | (allocated adj2 random).mp. | 293 |
| 34 | Single blind$.mp. | 22,288 |
| 35 | Double blind$.mp. | 128,916 |
| 36 | ((treble or triple) adj blind$).mp. | 310 |
| 37 | Placebo$.mp. | 247,083 |
| 38 | Prospective study/ | 239,458 |
| 39 | or/21-38 | 1,195,213 |
| 40 | case report.mp. | 1,132,346 |
| 41 | Abstract report/ or letter/ | 558,430 |
| 42 | exp case study/ | 21,434 |
| 43 | or/40-42 | 1,592,942 |
| 44 | 39 not 43 | 1,156,570 |
| 45 | 20 and 44 | 1,404 |
| 46 | limit 45 to yr="2012 -Current" | 272 |
