# Supplementary material for: Efficacy of Anti-VEGF and Laser Photocoagulation in the Treatment of Visual Impairment due to Diabetic Macular Edema: A Systematic Review and Network Meta-Analysis
Source: PLoS One. 2014 Jul 16;9(7):e102309. doi: 10.1371/journal.pone.0102309 (PMC4100770; doi:10.1371/journal.pone.0102309)
Supplement: Table S3 — Studies from randomized controlled trial database search excluded based on full text review. (DOCX) [file pone.0102309.s003.docx]

Table S3. Studies from randomized controlled trial database search excluded based on full text review.

| **Study** | **Reason for exclusion** |
| --- | --- |
| Nguyen QD *et al*. (2012) Dose-ranging evaluation of intravitreal siRNA PF-04523655 for diabetic macular edema (the DEGAS study) [[57](#_ENREF_57)] | siRNA not a relevant comparator |
| Ip MS *et al*. (2012) Long-term effects of ranibizumab on diabetic retinopathy severity and progression.[[56](#_ENREF_56)] | 24-month follow-up in RIDE and RISE |
| Elman MJ *et al*. (2012) Intravitreal ranibizumab for diabetic macular edema with prompt versus deferred laser treatment: Three-year randomized trial results.[[55](#_ENREF_55)] | 3-year follow-up in DRCR.net Protocol I |
| Do DV *et al*. (2013) Ranibizumab for edema of the macula in diabetes study: 3-year outcomes and the need for prolonged frequent treatment.[[54](#_ENREF_54)] | 3-year data from READ -2 |
| Yilmaz MB *et al*. (2012) Intravitreal bevacizumab for refractory diabetic macular edema.[[58](#_ENREF_58)] | No comparator of interest |
| Brown DM *et al*. (2013) Long-term outcomes of ranibizumab therapy for diabetic macular edema: the 36-month results from two phase III trials: RISE and RIDE.[[53](#_ENREF_53)] | 3-year data from RISE and RIDE |
| Bhavsar AR *et al*. (Diabetic Retinopathy Clinical Research Network) (2013) Randomized clinical trial evaluating intravitreal ranibizumab or saline for vitreous haemorrhage from proliferative diabetic retinopathy.[[52](#_ENREF_52)] | Only 16-week follow-up |
