# Supplementary material for: Efficacy of Anti-VEGF and Laser Photocoagulation in the Treatment of Visual Impairment due to Diabetic Macular Edema: A Systematic Review and Network Meta-Analysis
Source: PLoS One. 2014 Jul 16;9(7):e102309. doi: 10.1371/journal.pone.0102309 (PMC4100770; doi:10.1371/journal.pone.0102309)
Supplement: Table S4 — Quality appraisal of included systematic reviews. (DOCX) [file pone.0102309.s004.docx]

Table S4. Quality appraisal of included systematic reviews.

| **Study** | **Appropriate and clearly focussed question** | **Study methodology adequately described** | **Study literature searches adequate** | **Study quality assessed and taken into account** |
| --- | --- | --- | --- | --- |
| Ford 2013 [[30](#_ENREF_30)] | Well covered | Well covered | Well covered | Adequately addressed |
| Ollendorf 2013 [[33](#_ENREF_33)] | Adequately addressed | Adequately addressed | Adequately addressed | Adequately addressed |
| Virgili 2012 [[36](#_ENREF_36)] | Well covered | Well covered | Well covered | Well covered |
| Zechmeister-Koss 2011 [[32](#_ENREF_32)] | Adequately addressed | Adequately addressed | Adequately addressed | Adequately addressed |
