# Supplementary material for: Efficacy of Anti-VEGF and Laser Photocoagulation in the Treatment of Visual Impairment due to Diabetic Macular Edema: A Systematic Review and Network Meta-Analysis
Source: PLoS One. 2014 Jul 16;9(7):e102309. doi: 10.1371/journal.pone.0102309 (PMC4100770; doi:10.1371/journal.pone.0102309)
Supplement: Table S5 — Quality appraisal of included randomized controlled trials. (DOCX) [file pone.0102309.s005.docx]

Supplementary Table 5. Quality appraisal of included randomized controlled trials.

| Study | Randomization carried out appropriately | Adequate concealment of treatment allocation | Treatment groups similar at baseline in terms of prognostic factors | Care providers, participants and outcome assessors blind to treatment allocation | Any unexpected imbalances in drop-outs between groups | Any evidence to suggest that the authors measured more outcomes than they reported | Did analysis include an intention-to-treat analysis? If so, was this appropriate and were appropriate methods used to account for missing data? |
| --- | --- | --- | --- | --- | --- | --- | --- |
| DA VINCI [[66](#_ENREF_66)] | Yes* | Yes* | Yes | Yes (patients and outcome assessors) | No | No | Yes – full analysis set, patients received treatment and 1 outcome measurement |
| DRCR.net Protocol I [[67](#_ENREF_67)] | Yes | Yes | Yes | Yes for included arms (patients and outcome assessors) | No | No | Yes – ITT |
| RESTORE [[48](#_ENREF_48)] | Yes | Yes | Yes | Yes (patients and outcome assessors) | No | No | Yes – full analysis set, patients received treatment and 1 outcome measurement |
| READ-2 [[69](#_ENREF_69)] | Unclear | Unclear | Unclear | Unclear | No | No | Randomized set used |
| RESOLVE [[68](#_ENREF_68)] | Yes | Yes | Yes | Yes (patients, outcome assessors, evaluating investigators) | Yes | No | Yes – full analysis set, patients received treatment and 1 outcome measurement |

Conference abstracts and presentations were not assessed for their risk of bias due to limited study methodology being reported.

VIVID, VISTA, RESPOND (data on file).

* Based on the work by Virgili et al. (2012) [[36](#_ENREF_36)]. The authors contacted DA VINCI investigators who provided evidence of adequate randomization and allocation concealment.
