# Supplementary material for: Efficacy of Anti-VEGF and Laser Photocoagulation in the Treatment of Visual Impairment due to Diabetic Macular Edema: A Systematic Review and Network Meta-Analysis
Source: PLoS One. 2014 Jul 16;9(7):e102309. doi: 10.1371/journal.pone.0102309 (PMC4100770; doi:10.1371/journal.pone.0102309)
Supplement: Table S6 — Summary of baseline BCVA (ETDRS letter score) in the study eye by study and treatment group. (DOCX) [file pone.0102309.s006.docx]

Supplementary Table 6. Summary of baseline BCVA (ETDRS letter score) in the study eye by study and treatment group.

| **Study** | **Treatment group** | | | | |
| --- | --- | --- | --- | --- | --- |
|  | **Ranibizumab 0.5 mg PRN, Mean (s.d.)** | **Aflibercept 2.0 mg bi-monthly, Mean (s.d.)** | **Laser, Mean (s.d.)** | **Sham, Mean (s.d.)** | **Ranibizumab 0.5 mg PRN + laser, Mean (s.d.)** |
| DA VINCI [[66](#_ENREF_66)] |  | 58.8 (12.2) | 57.6 (12.5) |  |  |
| VISTA[[72](#_ENREF_72)] |  | 59.4 (10.9) | 59.7 (10.9) |  |  |
| VIVID[[72](#_ENREF_72)] |  | 58.8 (11.2) | 60.8 (10.6) |  |  |
| DRCR.net Protocol I[[67](#_ENREF_67)] |  |  | 65.0 (NR) |  | 66.0 (NR) |
| READ-2 [[69](#_ENREF_69)] | 24.9 (NR) |  | 28.4 (NR) |  | 24.9 (NR) |
| RESOLVE [[68](#_ENREF_68)] | 60.2 (9.9) |  |  | 61.1 (9.0) |  |
| RESPOND[[73](#_ENREF_73)] | 63.1 (10.6) |  | 61.9 (10.6) |  | 64.8 (9.3) |
| RESTORE [[48](#_ENREF_48)] | 64.8 (10.1) |  | 62.4 (11.1) |  | 63.4 (10.0) |

Not all treatment groups included in the network meta-analysis are presented. Analysis is based on the intention-to-treat population of each study.

BCVA, best-corrected visual acuity; bimonthly, every 2 months; ETDRS, Early Treatment Diabetic Retinopathy Study; NR, not reported; PRN, *pro re nata* (as needed); s.d.,, standard deviation.

Reported BCVA are the mean at baseline with the exception of DRCR.net Protocol I where only the median per arm was reported.
