# Supplementary material for: Efficacy of Anti-VEGF and Laser Photocoagulation in the Treatment of Visual Impairment due to Diabetic Macular Edema: A Systematic Review and Network Meta-Analysis
Source: PLoS One. 2014 Jul 16;9(7):e102309. doi: 10.1371/journal.pone.0102309 (PMC4100770; doi:10.1371/journal.pone.0102309)
Supplement: Table S7 — Summary of baseline CRT/CFT in the study eye by study and treatment group. (DOCX) [file pone.0102309.s007.docx]

TableS7. Summary of baseline CRT/CFT in the study eye by study and treatment group.

| **Study** |  | **Treatment Group** | | | | |
| --- | --- | --- | --- | --- | --- | --- |
|  | **Baseline characteristic** | **Ranibizumab 0.5 mg PRN, Mean (s.d.)** | **Aflibercept 2.0 mg bi-monthly, Mean (s.d.)** | **Laser, Mean (s.d.)** | **Sham, Mean (s.d.)** | **Ranibizumab 0.5 mg PRN + laser, Mean (s.d.)** |
| DA VINCI [[66](#_ENREF_66)] | CRT (μm) |  | 434.8 (111.8) | 440.6 (145.4) |  |  |
| VISTA[[72](#_ENREF_72)] | CRT (μm) |  | 479.0 (154.0) | 483.0 (153.0) |  |  |
| VIVID[[72](#_ENREF_72)] | CRT (μm) |  | 518.0 (147.0) | 540.0 (152.0) |  |  |
| DRCR.net Protocol I[[67](#_ENREF_67)] | CST (μm) |  |  | 407.0 (NR) |  | 371.0 (NR) |
| READ-2 [[69](#_ENREF_69)] | CFT (μm)* | 410.8 (NR) |  | 439.7 (NR) |  | 474.5 (NR) |
| RESOLVE [[68](#_ENREF_68)] | CRT (μm) | 455.4 (114.2) |  |  | 448.9 (102.8) |  |
| RESPOND[[73](#_ENREF_73)] | CRT (μm) | 448.5 (136.6) |  | 458.0 (133.1) |  | 422.1 (142.3) |
| RESTORE [[48](#_ENREF_48)] | CRT (μm) | 426.6 (118.0) |  | 412.4 (124.0) |  | 416.4 (119.9) |

Not all treatment groups included in the network meta-analysis are presented. Analysis is based on the intention-to-treat population of each study.

Bimonthly, every 2 months; CRT, central retinal thickness; CFT, central foveal thickness; CST, central subfield thickness; NR, not reported; PRN, *pro re nata* (as needed); s.d., standard deviation.

*READ-2 reports mean excess foveal thickness, calculated by subtracting 212 μm (upper limit of the normal range of centre subfield thickness) from the measured foveal thickness value. CFT values reported above are the excess foveal thickness data reported in READ-2, plus 212 μm.
