# Supplementary material for: Efficacy of Anti-VEGF and Laser Photocoagulation in the Treatment of Visual Impairment due to Diabetic Macular Edema: A Systematic Review and Network Meta-Analysis
Source: PLoS One. 2014 Jul 16;9(7):e102309. doi: 10.1371/journal.pone.0102309 (PMC4100770; doi:10.1371/journal.pone.0102309)
Supplement: Information S1 — Fixed and random treatment effect model. (DOCX) [file pone.0102309.s009.docx]

# Supporting information S1

***Fixed treatment effect model***

$r_{i,k}\sim dbin(p_{i,k},n_{i,k})$ [1]

logit$(p_{i,k})=\mu_{i}+d_{t(i,k)}+\beta\times BCVA_{i,k}-d_{t(i,b)}-\beta\times BCVA_{i,b}$ [2]

$d_{l}\sim N(0,1000)$ if *l*=aflibercept, ranibizumab, ranibizumab+laser and $d_{l}=0$if *l*=laser [3]

$\mu_{i}\sim N(0,1000)$ [4]

$\beta\sim N(0,1000)$ [5]

## Random treatment effect model (base case)

$r_{i,k}\sim dbin(p_{i,k},n_{i,k})$ [1]

logit$(p_{i,k})=\mu_{i}+\delta_{i,k}$ [6]

$\delta_{i,k}\sim N(d_{t(i,k)}+\beta\times BCVA_{i,k}-d_{t(i,b)}-\beta\times BCVA_{i,b},\sigma^{2})$ [7]

$d_{l}\sim N(0,1000)$ if *l*=aflibercept, ranibizumab, ranibizumab+laser and $d_{l}=0$if *l*=laser [3]

$\mu_{i}\sim N(0,1000)$ [4]

$\beta\sim N(0,1000)$ [5]$\sigma\sim dunif(0,2)$ [8]

Where is the number of patients who gained 10 letters in the *k^th^* arm of trial *i*, is the total number of patients in the *k^th^* arm of trial *i* and the percentage of patients gaining 10 letters the model estimates. *BCVA* is the baseline visual acuity*. t*(*i*,*k*) is a function that identifies the product in *k^th^* arm in trial *i* and *t*(*i*,*b*) is a function that identifies the product in the control arm in trial *i*. *t*(*i*,*k*) and *t*(*i*,*b*) can be one of take 4 values: laser, ranibizumab, ranibizumab+laser or aflibercept. *d_l_* can be interpreted as the incremental treatment effect of treatment *l* vs. laser. *β* represents the impact that one additional letter have on the odds that patient will gain 10 letters. Equations [4], [5] and [8] show that *β*, *σ* and $\mu_{i}$are assumed to follow uninformative priors.

In all models, the relative efficacy (odds ratio) of ranibizumab vs. aflibercept is estimated by the posterior distribution of exp(*d_ranibizumab_* - *d_aflibercept_*)

Note: The fixed and random treatment effect model adjusted for thee correlation between treatments effects in multi-arm trials (corrections not shown in above equations).
